# Understanding Why Children Commit Scale Errors: Scale Error and Its Relation to Action Planning and Inhibitory Control, and the Concept of Size

**DOI:** 10.3389/fpsyg.2017.00826

**Published:** 2017-05-23

**Authors:** Mikako Ishibashi, Yusuke Moriguchi

**Affiliations:** ^1^Department of Psychology, Ochanomizu UniversityTokyo, Japan; ^2^Graduate School of Education, Kyoto UniversityKyoto, Japan

**Keywords:** scale error, concepts of size, action planning, inhibitory control, young children

## Abstract

Scale error is a phenomenon where young children attempt to perform inappropriate actions on miniature object without considering the actual size of the object. The present study examined two hypotheses on what factors contribute to the occurrence of scale errors, focusing on the following possible factors: action planning and inhibitory control, and concept of size. Thus, we hypothesize that scale errors derived from either immaturity of their action planning and inhibitory control abilities or understanding of size concepts. The results revealed that the concept of size was significantly negatively associated with the occurrence of scale errors. However, action planning and inhibitory control were not significantly associated with the occurrence of scale errors. These results suggest that scale errors may arise from a misunderstanding of size concepts.

## Introduction

Young children sometimes attempt to perform inappropriate actions without considering the size of an object. They deliberately try to fit their bodies onto/into extremely tiny objects, such as trying to ride on a miniature car or chair. This phenomenon is called a scale error (DeLoache et al., 2004). In the original study by DeLoache et al. (2004) about half of 18- to 30-month-old children committed at least one scale error. Scale errors were most often seen in children at around 2 years of age and characterized by showing the inverted U-shape function of age. Recent studies have shown that the scale error is a robust phenomenon that can be observed in daily life. Indeed, children commit scale errors in experimental situations, in preschool classrooms, and at home when playing with miniature objects, such as a doll or tool (DeLoache et al., 2004, 2013; Ware et al., 2006, 2010; Brownell et al., 2007; Rosengren et al., 2009; Casler et al., 2011).

Although the previous studies demonstrate that children commit scale errors (DeLoache et al., 2004, 2013), few experimental research thus far has examined potential factors that involved in the production of scale errors. The present study aimed to experimentally examine what factors contribute to the occurrence of a scale error. We focused on two hypotheses that point to the factors of action planning and inhibitory control, and the concept of size, respectively. The goal was to identify which hypothesis provides the most plausible explanation for children’s scale errors.

As the hypotheses of action planning and inhibitory control, DeLoache et al. (2004) proposed that the mechanism behind scale errors involves dissociation between visual information for planning and for executing actions. In their study, children’s mental representation of the general or familiar size of the object was activated by seeing a miniature object. The process also includes the activation of a motor plan that is associated with the general category of the object (Ware et al., 2006). In general, the activated motor plan is inhibited by the registration of actual visual information about the miniature object (DeLoache et al., 2004; Ware et al., 2006). However, for young children, the information about the size of the miniature object may not inhibit the activated motor plan for the general object (Ware et al., 2006; DeLoache and Uttal, 2011). Thus, children’s mental representations may identify the miniature object as part of the class of such objects, which includes information about the particular or general size, but the representations may not include the specific visual information about the miniature object, such as its tiny size (DeLoache and Uttal, 2011). Therefore, the children may fail to inhibit the motor plan for the general object and may formulate an inappropriate action plan based on the usual size of the general class of objects that is activated by the miniature object. In this explanation, children commit scale errors because of the immaturity of their inhibitory control and action planning ability. Thus, children who are better at inhibitory control and action planning abilities may be less likely to exhibit scale errors.

According to the second hypothesis, another possible factor that may affect children’s scale errors is the concept of size. There might be two possible effects of this concept on scale errors. On the one hand, conceptual development at around 2 years of age may lead to the incidence of scale errors. DeLoache and Uttal (2011) suggested that children’s immature conceptual representation is not strong enough to activate the relevant category of the miniature object. Infants’ conceptual representations become more sophisticated at around 2 years of age, and the associated motor routines become well-integrated. This integration may induce the overriding of the perception of the object’s actual size, leading to a scale error. Conversely, it is equally possible that conceptual development may suppress the occurrence of scale errors. As mentioned above, scale errors may arise from the identification of the object that does not include information about the specific miniature in sight, such as its tiny size (DeLoache and Uttal, 2011). Some studies have investigated that children fail to perform fit in an appropriate spatial relations, indicating children may fail to understand the size relations between object and other objects (Shutts et al., 2009). In other words, the conceptual development may facilitate the distinction between the miniature and general sizes of the object, which may lead to the suppression of scale errors. Thus, children who are better at understanding size concepts may be less likely to exhibit scale errors.

To test each of the two hypotheses, we employed an appropriate task. For the action planning and inhibitory control hypothesis, we used a bar task, posting task and an A-not-B task. The bar task requires planning to select the most efficient plan in accordance with the direction of an object (Jovanovic and Schwarzer, 2011). Jovanovic and Schwarzer (2011) reported that it is not until the age of 3 that children were able to integrate the different step into an action plan. The posting task requires children to use planning ability to match an object with an appropriately sized hole in advance (Street et al., 2011). Street et al. (2011) reported that 18-month-olds failed to match the objects properly, but 24-month-olds succeeded in the task, suggesting a dramatic change in planning ability between 18 and 24 months. The A-not-B task has been used to index inhibition process for infants and young children (Diamond and Goldman-Rakic, 1989; Espy et al., 1999; but see also Munakata, 1998; Smith et al., 1999). In this task, the children need to search a new location to find a desired object instead of searching a previously successful location. Although the original version of the task was for infants around 1 year of age, Espy et al. (1999) used a modified version of the A-not-B tasks and found that children’s performance on the A-not-B task improved between 23 and 66 months of age.

We also examined the relation between the concept of size and scale error. We assessed the children’s concepts using a parent questionnaire, the Kinder Infant Development Scale (KIDS). KIDS is widely used to assess children’s cognitive development from 0 to 6 years old (Miyake et al., 1989; Cheng et al., 2009; Negayama et al., 2010; Hashimoto et al., 2013). We used “language concepts” to examine whether children understood the concept. These items included measures of children’s concept of size, such as understanding big and small (i.e., “Can your child understand big and small”, “Can your child understand wide and narrow”).

## Methods

### Participants

The participants were 54 children (*M* = 24.0 months, *SD* = 5.4) and their parents. Two additional children participated but were not included in final sample due to fussiness. The sample size was determined based on a previous study that examined the relation between the scale error task and other tasks (Brownell et al., 2007; *N* = 57). The children were divided into four age groups: 21 children aged 16–20 months (*M* = 19.0 months, *SD* = 1.2, 15 males, six females), 14 children aged 21–25 months (*M* = 23.0, *SD* = 1.6, seven males, seven females), 14 children aged 26–31 months (*M* = 28.0, *SD* = 1.9, eight males, six females), and five children aged 32–37 months (*M* = 35.0, *SD* = 1.5, two males, three females). These age ranges were selected based on a previous study (DeLoache et al., 2013: Experiment 3). Among the 54 participants, the data of one child’s bar task was excluded due to fussiness. We recruited five children aged 32–37 months because we predicted children less than 31 months of age were more likely to commit scale errors (Ware et al., 2006). The inhibitory control task was conducted by 29 children because the stimuli have not been prepared at the time of the research project. The four age groups were as follows: 12 children aged 16–20 months (*M* = 19.33 months, *SD* = 1.15, 10 males, two females), seven children aged 21–25 months (*M* = 23.29, *SD* = 1.5, four males, three females), eight children aged 26–31 months (*M* = 28.13, *SD* = 1.73, five males, three females), and two children aged 32–37 months (*M* = 36.0, *SD* = 0, one male, one female).

We examined the age difference between children who were given the inhibitory control task and were not. Independent samples *t*-tests revealed that there was no significant difference in age between both groups [*t*(52) = 0.306, *p* > 0.10, *d* = 0.090].

The participants were recruited from a registry of families maintained in the Child Development Lab at Joetsu University of Education. The parents provided written informed consent for the children to participate and were verbally informed about the purpose of the study. The experiment was approved by the ethics committee at Joetsu University of Education.

### Materials and Procedure

The experiments were conducted in a laboratory room at Joetsu University of Education. During the experiments, the children were free to interact with their parents. The experiments were videotaped. All the children were given the posting, bar, and scale error tasks. Afterward, 29 children received the inhibitory control task. The experiments were conducted in the following fixed order, (1)–(4). The parents were engaged in questionnaire (5).

#### (1) The Posting Task

We used the posting task developed by Street et al. (2011). The materials were a box and disk, both were composed of the same type of cardboard. The box was 28 cm × 40 cm × 10 cm. The disk was 8 cm in diameter and 0.8 cm in thickness. There was a hole (10 cm × 1 cm) in the center of the box that was large enough for the disk to be inserted.

The experimenter placed the box in front of the child. She demonstrated how to insert the disk into the hole and then instructed the child, “Now you try it.” The direction of the box was horizontally or vertically changed in each trial by the experimenter. We observed whether the child would change the direction of the disk in accordance with the direction of the box. In this task, the children needed to use action planning to adjust their action according to the direction of the hole. There were six trials, and the order of direction was random across participants.

#### (2) The Bar Task

**FIGURE 1:**
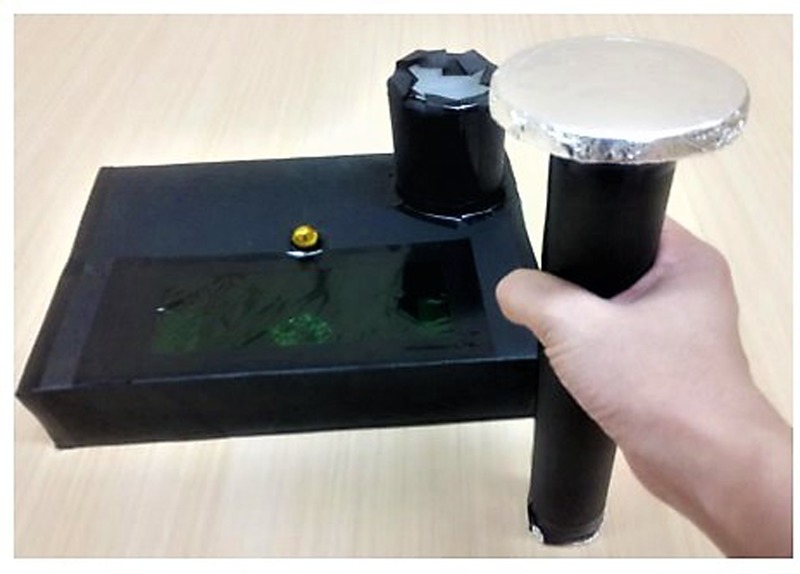
**The bartask**.

We used the bar task developed by Jovanovic and Schwarzer (2011). The materials were a bar and box, both were composed of cardboard. The box had a plastic cylinder attached. The bar was 26 cm long, with one broad end (6.5 cm diameter) and one narrow end (4 cm diameter). The plastic cylinder was 6.5 cm in diameter and 8.5 cm in height, and it was attached in the upper right corner of the box (21 cm × 27 cm × 5 cm). The broad side of the bar had the same diameter as the plastic cylinder; therefore, it did not fit into the cylinder. Thus, the size of the cylinder was fitted with only the narrow side of the bar. Two miniature bulbs were made to protrude out by inserting the bar into the cylinder. The bar task is shown in **Figure 1**.

At first, the experimenter placed the bar straight up, with the side of the narrow end touching the floor. This allowed the experimenter to grasp it without having to reorient the direction. The experimenter lifted the bar and inserted it into the cylinder. The event was modeled once to the children. Then, the bar was returned to the first position. The experimenter instructed the children, “Now you try it.” After that, the experimenter reversed the bar so that the side of the broad end touched the floor. To insert the bar smoothly into the cylinder, the children had to reverse the bar by hand in advance. We observed whether the children changed their grasp in accordance with the direction of the bar. In this task, action planning was needed to select proper grasping in accordance with the direction of the bar. The tasks comprised three trials.

#### (3) The Scale Error Task

The scale error task was based on DeLoache et al. (2004). The materials used were a slide, a car, a desk set (desk, chair, and a book), and shoes. Each object comprised a child-sized object and a miniature replica; however, for the shoes, we used only a miniature version because the children put on and removed their own shoes before engaging in the tasks. For the book, we placed a picture book on the desk to prompt the children to sit in the chair. The child-sized objects were three or four times the size of the miniature objects. The dimensions of the child-sized and miniature objects, respectively, were as follows: slide, 46 cm × 110 cm × 72 cm and 5 cm × 21 cm × 14 cm; car, 58 cm × 37.5 cm × 35.5 cm and 7 cm × 6 cm × 10 cm; desk set, 61 cm × 41 cm × 47.5 cm and 10 cm × 18 cm × 15 cm; chair, 35.5 cm × 28.5 cm × 32 cm^3^ and 8 cm × 10 cm × 10 cm; book, 12 cm × 17 cm and 2 cm × 3 cm; shoes, 4 cm × 7 cm × 2 cm (miniature only).

The slide, desk set, and car were placed in the play room. In the task, the children freely played with them for approximately 5 min. If he or she showed little interest in an object, the experimenter drew attention to it. The child was encouraged to interact with each toy at least twice. After that, the child and his or her parent left the play room. Meanwhile, the experimenter replaced the toys with their miniature versions and added the miniature shoes. Then, the child and his or her parent entered the room again. We observed the child’s behavior for approximately 5 min.

#### (4) The Inhibitory Control Task (the Modified A-not-B Task)

The materials for the inhibitory control task were two green cups, a tray, and a toy airplane. Each cup was 8 cm in diameter and 8 cm in height. The dimensions of the tray were 20 cm × 28.5 cm × 6 cm. The dimensions of the toy airplane were 6 cm × 6 cm × 3 cm. These materials were modeled on a task that had previously been used by Espy et al. (1999).

The experimenter and the child sat down at the table across from each other. The experimenter gave the child the toy airplane, and the child played with it for several seconds. After that, the experimenter asked for the toy back and hid it under one of the two cups on the tray while the child watched. After a few seconds, the experimenter asked, “Which cup is the toy hidden under?” After the child picked up one of the two cups, the experimenter returned the tray to the first position. We observed whether the child searched under the appropriate-sized cup by inhibiting a search of the previous cup. There were six trials.

#### (5) Language concepts

KIDS questionnaire (Miyake et al., 1989) were used to examine children’ language concepts. Language concepts comprises 13 items for children 12–35 months of age (for children aged 3–6 years, there are 16 items). The items were rated on a 2-point scale (0 = definitely not or not sure, 1 = definitely).

### Data Coding and Analysis

#### (1) The Posting Task

We divided the children’s actions into three groups as follows: failure, success after failure, and success. Failure indicated that the child could not fit the disk into the hole. Success after failure indicated that the child succeeded in inserting the disk after initially trying to fit it in with the wrong orientation. Success indicated that the child fit the disk in on the first try. The score was 0 for failure, 1 for success after failure, and 2 for success. We computed each child’s overall accuracy by taking the average of the six trials. Twenty-five percent of the sample was recorded by the secondary coder, and interrater reliability was measured using Cohen’s kappa (κ = 0.92).

#### (2) The Bar Task

Based on Jovanovic and Schwarzer (2011), we divided the children into five groups according to their grasping: children who did not insert the bar into the cylinder; those who inserted it after initially trying to insert it with the wrong orientation; those who inserted it using both hands; those who inserted it using one hand, by grasping the bar with a thumb-up grip; and those who inserted it using one hand by grasping the bar with a thumb-down grip. Each group was given from 1 to 5 points. We computed the overall accuracy by averaging the results of the three trials. Twenty-five percent of the sample was recorded by the secondary coder, and the interrater reliability was measured using Cohen’s kappa (κ = 0.91).

#### (3) The Scale Error Task

We adopted the coding criteria used by DeLoache et al. (2013): (a) The child attempted to perform part or all of the action as if he or she were using the child-sized object. (b) The child touched an appropriate body part(s) to the appropriate part(s) of the miniature object, for example, the child’s foot touched the slide’s stairs. (c) The child’s effort (facial expression) was serious, not pretending. (d) The child continued the action relatively persistent. Children were regarded as committing a scale error if the children met the four criteria.

In regards to (c), we coded the seriousness of the children’s attempts using a 5-point scale: 1 (definitely serious), 2 (probably serious), 3 (not clear), 4 (probably pretending), and 5 (definitely pretending). We regarded the children’s attempts that were coded 1 or 2 as scale errors. Multiple actions toward a target object were counted as a single scale error. For example, if the children stepped on the stairs of the slide and then sat on the slide, we counted the behavior as one scale error. If the children committed a scale error toward the slide, sat in the chair, and then committed a scale error on the slide again, we counted two scale errors toward the slide. Twenty-five percent of the sample was recorded by the secondary coder, and interrater reliability was measured using Cohen’s kappa (κ = 0.91).

#### (4) The Inhibitory Control Task

A score of 1 was given when the child selected the correct cup, and a score of 0 was given for incorrect responses. We computed the overall accuracy by averaging the six trials. Twenty-five percent of the sample was recorded by the secondary coder, and the interrater reliability was measured using Cohen’s kappa (κ = 0.88).

#### (5) Language concepts

Parents completed the KIDS during their children engaged in tasks. We used the items related to language concepts for the analysis. On 13 of the 122 items was language concepts (for children aged 3–6 years, 16 of the 133 items was language concepts). We computed the total scores (range 1–29) by adding two scores for children 12–35 months of age (range 1–13) and children aged 3–6 years (range 14–29).

## Results

First, we report the descriptive results for all the variables in this study. Then, the contact time of each object during the scale error task are shown. Finally, the relation among variables are reported.

### Descriptive Statistics

First, we describe the number of scale errors. Twenty-six out of the 54 children performed scale errors. The number of scale errors was as follows: 2.83 (*SD* = 2.98) for the children aged 16–20 months, 3.29 (*SD* = 1.90) for the children aged 21–25 months, 2.38 (*SD* = 0.79) for the children aged 26–31 months, and 0 for the children aged 32–37 months. A one-way analysis of variance (ANOVA) was conducted to examine the age-related changes in the number of scale errors among the groups. We excluded the data of the 32- to 37-month-old children from the one-way ANOVA because they did not commit scale errors. We also did not include the effect of gender because there was no main effect and interaction. A one way ANOVA revealed that there was no significant main effect of age [*F*(2,46) = 0.12, *p* > 0.10, η_p_^2^ = 0.27].

The mean scores of the posting task were as follows: 1.33 (*SD* = 0.51) for the children aged 16–20 months, 1.80 (*SD* = 0.33) for the children aged 21–25 months, 1.93 (*SD* = 0.19) for the children aged 26–31 months, and 2.00 (*SD* = 0.58) for the children aged 32–37 months. A one-way ANOVA was conducted to examine the age-related changes in the mean scores of the posting task between groups. We found a significant main effect of age [*F*(3,50) = 9.72, *p* < 0.001, η_p_^2^ = 0.136]. A *post hoc* analysis using Tukey’s honest significant difference (HSD) test showed that the participants aged 16–20 months produced significantly worse scores than the other three age groups (*p* < 0.05). No other differences were significant.

Next, the mean scores of the bar task were as follows: 1.82 (*SD* = 0.95) for the children aged 16–20 months, 2.54 (*SD* = 1.21) for the children aged 21–25 months, 2.62 (*SD* = 0.76) for the children aged 26–31 months, and 2.8 (*SD* = 1.43) for the children aged 32–37 months. We conducted a one-way ANOVA to reveal the effect of age on the mean scores of the bar task among the groups. The main effect was not significant for age [*F*(3,48) = 2.55, *p* > 0.10, η_p_^2^ = 0.02].

The mean scores of the inhibitory control task were as follows: 4.25 (*SD* = 1.22) for the children aged 16–20 months, 4.57 (*SD* = 1.9) for those aged 21–25 months, 4.62 (*SD* = 1.51) for those aged 26–31 months, and 5 (*SD* = 1.97) for those aged 32–37 months. A one-way ANOVA was conducted to examine the age-related changes in the mean scores of the inhibitory control task among the groups. There was no significant main effect of age [*F*(3,25) = 0.22, *p* > 0.10, η_p_^2^ = 0.001].

Finally, the mean scores of language concepts were as follows: 5.76 (*SD* = 2.76) for the children aged 16–20 months, 7.93 (*SD* = 3.96) for the children aged 21–25 months, 9.21 (*SD* = 2.26) for the children aged 26–31 months, and 18.20 (*SD* = 4.60) for the children aged 32–37 months. A one-way ANOVA was conducted to examine the age-related changes in the mean scores of the language concepts between groups. There was a significant main effect of age [*F*(3,50) = 22.18, *p* < 0.001, η_p_^2^ = 0.57]. A *post hoc* analysis using Tukey’s HSD showed that the participants aged 32–37 months produced significantly higher scores than the other three age groups (*p* < 0.001). No other differences were significant.

### Correlations

Pearson partial correlations were calculated to reveal the relation between the number of scale errors and the other variables after controlling for age. We found a significant negative relation between the language concepts and the number of scale errors (*r* = -0.34, *p* < 0.05). In addition, the posting task and the bar task were significantly and negatively correlated (*r* = -0.61, *p* < 0.001; **Table 1**). No other significant effects were found. The correlation between the language concepts and the number of scale errors, and the posting task and the bar task remained significant using false discovery rate (FDR) correction (Benjamini and Hochberg, 1995). In addition, the partial correlations were calculated between the inhibitory control task and the number of scale errors after controlling for age because about half of the children did not participate in the inhibitory control task. There was no significant relation between the number of scale errors and the inhibitory control task (*r* = -0.20, *p* > 0.10).

**Table 1:** Partial correlation among tasks and language concepts for KIDS.

	2	3	4
1 Number of scale errors	-0.34^∗^	-0.14	-0.12
2 Language concepts		-0.08	-0.01
3 Posting task			0.61^∗∗∗^
4 Bar task			

*N = 53. ^∗^*p* < 0.05. ^∗∗∗^*p* < 0.001.*

### Regression Analyses

Hierarchical regression analyses were conducted to examine the relation between the number of scale errors and the language concepts. We entered age at Step 1, and then entered the posting task, bar task, and language concepts at Step 2 as potential predictors of the number of scale errors (see **Table 2**). We excluded the performances of an inhibitory control task from the analysis because about half of the children did not participate in the inhibitory control task.

**Table 2:** Hierarchical regression models of age, language concepts, the posting task, and the bar task.

	*B*	*SE B*	β^∗^
**Step 1**			
Age	-0.07	0.05	-0.20
**Step 2**			
Age	0.09	0.08	0.27
Language concepts	-0.11	0.04	-0.50^∗^
Posting task	-0.76	0.87	-0.18
Bar task	-0.05	0.31	-0.03

*R^2^ = 0.042 for Step 1; *R*^2^ = 0.180 (*p* < 0.05) for Step 2. ^∗^*p* < 0.05.*

At Step 1, age was not a significant predictor of the number of scale errors (*b* = -0.07, *SE b* = 0.05, *b*^∗^ = -0.20, *p* > 0.10, *R*^2^ = 0.04). At Step 2, we found that the posting task (*b* = -0.76, *SE b* = 0.87, *b*^∗^ = -0.18, *p* > 0.10, *R*^2^ = 0.18) and bar task (*b* = -0.05, *SE b* = 0.31, *b*^∗^ = -0.03, *p* > 0.10, *R*^2^ = 0.18) were not significant predictors of the scale errors. However, language concept was a significant predictor (*b* = -0.11, *SE b* = 0.04, *b*^∗^ = -0.50, *p* < 0.02, *R*^2^ = 0.18).

## Discussion and Conclusion

The present study tested the hypotheses that the occurrence of scale errors was associated with action planning and inhibitory control, and the concept of size. The results revealed that the performances of the planning and inhibitory control tasks were not significantly related to the occurrence of scale errors. On the other hand, the language concepts significantly predicted the occurrence of scale errors. The results revealed that the performance of the planning task and the A-not-B error task did not relate to the occurrence of scale errors. In the present study, we hypothesized that children failed to inhibit the motor plan for the general category of the object, and instead they executed the inappropriate action plan (the miniature objects), leading to a scale error. The previous research has suggested that children who committed scale errors attempted to perform inappropriate actions toward miniature objects, but their movements were accurately controlled toward the miniature objects (DeLoache et al., 2004). For instance, when children tried to open the miniature car’s door, they were able to adjust their hands based on its size. This may be due to the fact that children have at least two action plans that involve the general category and the miniature object, respectively. Several studies have also indicated that formulating the action plan and execution of movement are processed differently in infants (McCarty et al., 2001; Claxton et al., 2003; Von Hofsten, 2004). For instance, Claxton et al. (2003) reported that before 12 months, infants’ motor control was affected by the representation of a future state of events, not immediate perceptual features in motor planning tasks With growing age, infants are able to integrate the object properties (i.e., objects orientation and its size) into motor control (von Hofsten and Rönnqvist, 1988). This finding may provide further empirical support that the occurrence of scale errors is reflected the immaturity of anticipated action planning, not of motor control. Alternatively, the failure to obtain the significant relationship between the planning tasks and the scale error may be due to that motor function assessed in the planning tasks may be different from the motor function in scale errors. The planning task may be related to gross motor function whereas the production of scale errors may be related to fine motor skill. Furthermore, the performance on the A-not-B task did not relate to the occurrence of scale errors. The results did not support the hypotheses that scale errors might be caused by the immaturity of the children’s inhibitory control. We assumed that children failed to inhibit the motor plan for the general category of the object, and instead they executed the action plan for the miniature objects. This finding contradicted the idea by DeLoache et al. (2004) that scale errors stems from the immaturity of children’s inhibitory control. Nevertheless, it is possible that the planning and inhibitory control tasks used in the present study may be too easy for older children, producing a ceiling effect. Previous studies have shown that inhibitory control rapidly develops during preschool years, and several tasks are used to assess its development (Gerstadt et al., 1994; Moriguchi, 2012). Therefore, further research should be performed to examine the validity of the results using other action planning and inhibitory control tasks.

We found a significant relation between the occurrence of scale errors and the concept of size. We assumed two possible explanations regarding the concept of size. One possibility was that the development of the object concept might lead to a scale error. The other was that the lack of an object concept may lead to a scale error. Our results supported the latter possibility. Thus, the scale error may be due to the failure to identify a miniature object as miniature (DeLoache and Uttal, 2011). However, one limitation of this finding was its reliance on a single informant measurement (the language concepts of KIDS) of the concepts of size. This issue should be examined by using multiple tasks to assess the concepts.

We found no significant main effect of age in the number of scale errors among the groups. Some previous studies showed that the incidence of scale errors showed the inverted U-shape function of age (DeLoache et al., 2004). Although we did not replicate the results, our results showed the similar developmental pattern.

In conclusion, this study suggested that scale errors stemmed from the failure of concepts of size, and not from poor action planning and inhibitory control. Despite the above limitations, this study contributes to our understanding of the mechanism of scale errors. Future studies should examine the extent to which these results are robust across different participant samples and measures.

## Author Contributions

YM and MI: Substantial contributions to the conception or design of the work; or the acquisition, analysis, or interpretation of data for the work. YM and MI: Drafting the work or revising it critically for important intellectual content. YM and MI: Final approval of the version to be published. YM and MI: Agreement to be accountable for all aspects of the work in ensuring that questions related to the accuracy or integrity of any part of the work are appropriately investigated and resolved.

## Conflict of Interest Statement

The authors declare that the research was conducted in the absence of any commercial or financial relationships that could be construed as a potential conflict of interest.
